# Explainable CNN for brain tumor detection and classification through XAI based key features identification

**DOI:** 10.1186/s40708-025-00257-y

**Published:** 2025-04-30

**Authors:** Shagufta Iftikhar, Nadeem Anjum, Abdul Basit Siddiqui, Masood Ur Rehman, Naeem Ramzan

**Affiliations:** 1 Department of Computer Science, Capital University of Science and Technology, Islamabad, Pakistan; 2 James Watt School of Engineering, University of Glasgow, Glasgow, G12 8QQ UK; 3 School of Computing, Engineering and Physical Sciences, University of the West of Scotland, Paisley, PA1 2BE UK

**Keywords:** Convolutional neural network, Deep learning, Explainable AI, Brain tumor classification

## Abstract

Despite significant advancements in brain tumor classification, many existing models suffer from complex structures that make them difficult to interpret. This complexity can hinder the transparency of the decision-making process, causing models to rely on irrelevant features or normal soft tissues. Besides, these models often include additional layers and parameters, which further complicate the classification process. Our work addresses these limitations by introducing a novel methodology that combines Explainable AI (XAI) techniques with a Convolutional Neural Network (CNN) architecture. The major contribution of this paper is ensuring that the model focuses on the most relevant features for tumor detection and classification, while simultaneously reducing complexity, by minimizing the number of layers. This approach enhances the model’s transparency and robustness, giving clear insights into its decision-making process through XAI techniques such as Gradient-weighted Class Activation Mapping (Grad-Cam), Shapley Additive explanations (Shap), and Local Interpretable Model-agnostic Explanations (LIME). Additionally, the approach demonstrates better performance, achieving 99% accuracy on seen data and 95% on unseen data, highlighting its generalizability and reliability. This balance of simplicity, interpretability, and high accuracy represents a significant advancement in the classification of brain tumor.

## Introduction

A tumor forms when cells grow abnormally and aggregate into a mass or lump, significantly representing deviating from normal cellular behavior. Unlike healthy cells, which grow, divide, and die in an orderly manner, tumor cells disrupt this process. Brain tumor is identified as one of the most life-threatening diseases globally. Various factors contribute to brain tumor development, including air pollution as a significant external factor [1] and genetic variation, which accounts for 5-10% of cases, especially with a family history. Additionally, radiation exposure in the workplace also increases risk [2]. Therefore, understanding the brain structure and function is crucial for identifying and treating brain tumors [3]. The brain structure consists of three primary regions: the cerebrum, the cerebellum, and the brain stem. The cerebrum, in charge of conscious thinking, behavior, and motion, is separated into two hemispheres, which are linked by the corpus callosum, and is sectioned into four lobes, each in charge of varied tasks. The cerebellum, positioned under the cerebrum, is vital for maintaining balance, coordination, and processing signals from the cerebral cortex through its three-layered cerebellar cortex. The brain stem links the spinal cord and brain, including the midbrain, pons, and medulla oblongata. It regulates essential bodily functions and transmits messages between the brain and organs. The categorization of the brain in terms of tissues includes grey and white matter. Grey matter, found in the brain’s outermost layer (cerebral cortex), is formed by neuron cell bodies that process information [2]. White matter, located deeper, contains myelin-covered axons that connect brain regions and transmit signals throughout the brain and body. Brain tumors are categorized as benign (non-cancerous) and malignant (cancerous) categories. Benign tumors don’t progress or spread, and recurrence after removal is rare [4, 12]. Malignant tumors, however, spread rapidly and cause significant dysfunction without prompt treatment [4]. According to the WHO, there exist four grade classifications of brain tumors. Grades 1 and 2 refer to lower-grade (benign) tumors, such as meningioma and pituitary tumors. In contrast, grades 3 and 4 indicate more severe (malignant) tumors, such as gliomas. These tumors vary in location, shape, texture, and size, making classification challenging [14, 15] as shown in Table 1. Meningiomas often cause mild symptoms like morning migraines and visual disturbances. Pituitary tumors may compress the optic nerve, causing migraines, vision issues, and double vision. Gliomas can lead to symptoms such as aphasia, cognitive decline, visual impairment, balance problems, and more [12].

**Table 1:** Characteristics of brain tumor types

Type	Meningioma	Pituitary	Glioma
Image			
Characteristics	Extra-axial tumors develop adjacent to the meninges, which are the protective layers surrounding the brain and spinal cord	An abnormal growth can form in the pituitary gland, a small organ at the base of the brain responsible for hormone production	Intra-axial tumors often exhibit thick, irregular borders that enhance around a central necrotic core with hemorrhagic features, arise from the brain’s glial cells, which support neurons, and can emerge throughout the brain

These tumors are identified and classified by radiologists using different modalities of medical imaging. CT scans and MRIs are often adopted to acquire detailed information about the human body. MRI is preferred for brain tumor assessment due to its non-invasiveness, ability to generate detailed 3D images, and effectiveness in automated medical image analysis [16–19]. Radiologists analyzing brain MRI focus on distinguishing tumors from healthy tissue and categorizing tumor types [4]. Manual detection is hindered by diverse tumor shapes, sizes, and similar appearances among types, compounded by a lack of expertise, making it laborious and error-prone with large datasets [13, 20]. To enhance accuracy and efficiency in brain tumor classification, automated systems are critically needed [4].

To address this, several approaches have been developed to brain tumor detection and classification, incorporating machine learning (ML) and deep learning (DL) approaches. Traditional ML methods, such as Support Vector Machines (SVM), have been widely used due to their effectiveness in binary classification tasks and robustness against overfitting. SVM models, particularly Twin Support Vector Machines (TWSVM), have shown promise in brain tumor classification by effectively separating different tumor types using hyperplanes. However, conventional SVMs and TWSVMs often struggle with noisy data and require solving complex quadratic programming problems, affecting computational efficiency.

However, the superior performance of DL frameworks over conventional ML models in the tasks of image classification and object detection has highlighted their significance. Using multi-layer neural networks, it autonomously identifies complex patterns from images, enhancing accuracy and diagnostic efficiency, thereby revolutionizing medical imaging and diagnostics. Recent research has increasingly employed DL for the tasks of detection and classification of brain tumor compared to traditional approaches [4–7], motivating focused development in this area. However, DL’s black box nature, with complex neural network layers, poses challenges in interpreting decision-making processes despite its effectiveness. The complexity of DL networks can produce models that are challenging to understand. In medical settings, transparency in the decision-making process of automated systems is paramount. Understanding the logic behind a model’s diagnosis or treatment recommendation is essential for both healthcare professionals and patients [8–11]. Without effective explainability methods, these models might focus on non-discriminative or irrelevant features, such as normal soft tissues, instead of tumor areas, undermining the reliability of their classifications and potentially leading to incorrect diagnoses. Additionally, many existing models use complex architectures to enhance accuracy, but this increased complexity raises computational costs, making real-time clinical implementation more challenging. This is where Explainable AI (XAI) comes in, referring to the techniques and approaches used to analyze and interpret how these complex models arrive at their decisions. XAI aims to provide an understanding that how DL models reach the predictions or classifications, thereby enhancing transparency, and accountability.

In recent years, several DL approaches with XAI have been developed. However, these studies often lacked clear interpretability, resulting in inadequate explainability. This lack of interpretation causes models to focus on unnecessary features or normal soft tissues, making the entire system questionable. Additionally, brain tumor classification models often have additional layers and parameters, increasing their complexity. Simplifying a model reduces parameters, layers, or computational complexity, enhancing interpretability and deployment efficiency but potentially lowering feature extraction and classification accuracy. The challenge is balancing complexity and performance, as complex models may depend on irrelevant features, while simpler ones may struggle with complex patterns. Our method uses explainable AI (XAI) to maintain high accuracy by focusing on the most relevant tumor regions. Moreover, previous studies did not use XAI to simplify brain tumor classification models or enhance their performance. Therefore, our objective is to use XAI to develop an interpretable system that can improve the model’s architecture and behavior, thereby enhancing overall performance.

The prime contributions of our research include:

1. Identifying real contributing abnormal tissues from the brain MRIs using XAI to enhance the overall performance of brain tumor classification.
2. Achieving an interpretable model that provides transparency into model decision-making process making it superior to existing non-interpretable approaches.
3. Providing insights into the decision-making process of model to reduce the number of layers in brain tumor classification models.
4. Achieving improved accuracy, precision, recall, and F1-score of 99% on Dataset-1 and 94% on Dataset-2.

This research paper is organized as follows: Sect.  conducts a detailed literature review of brain tumor classification using DL approaches with and without XAI. Section  details the employed methodology, focusing on the utilization of XAI to make the brain tumor classification model less complex and more interpretable. Section  details the results and discusses their significance. Lastly, section draws the study’s conclusions and outlines promising future directions for research.

## Related Works

Various researchers have proposed approaches for brain tumor classification. The inclusion criteria for our research for selecting literature review papers were based on their relevance to deep learning brain tumor classification using MR images. Moreover, papers on brain tumor classification with explainability are also selected. All these selected papers are from the years 2022 and above.

**Table 2:** Analysis of Existing Techniques Based on Deep Learning with and without XAI

Method	Dataset (MRIs)	Accuracy	XAI Method	Limitation
Conv Atten Mixer [21]	7023	97%	–	Complex architecture
CNN-7 Layers [22]	253	94%	–	4.4cmLimited dataset & performed Binary Classification
AlexNet-KNN [23]	2880	97%	–	Limited dataset
Image Enhancement + Modified CNN [24]	7023	97%	–	Complex architecture
Modified CNN [25]	7023	96%	–	Achieved low accuracy
InceptionV3 [26]	7023	97%	–	Complex architecture
CNN-24 Layers [27]	3264	94%	–	Achieved low accuracy
VGG-CNN [28]	4200	96%	–	Complex architecture
MobileNet [29]	7023	99%	–	Complex architecture
CNN-KNN [30]	2879	95%	–	Achieved low accuracy
Twin SVM with fuzzy hyperplane [31]	15 datasets	93%	–	Achieved low accuracy
CNN [32]	3 datasets	–	–	Result not found
ViT [33]	3064	98%	–	High computational cost
DenseNet [34]	3064	97%	–	Limited Dataset
VGG19 [35]	253	98%	Grad-Cam	Limited dataset & Performed Binary Classification
VGG16 [36]	3000	N/A	Shap	Performed Binary Classification & Results not found
VGG16 [37]	3000	97%	LRP	Limited dataset & Performed Binary Classification
CNN with dual-input [38]	2870	85%	Lime and Shap	Limited dataset & Achieved low accuracy
Resnet50 [39]	3000	99%	Grad-Cam	Limited dataset & Performed Binary Classification

### Deep learning techniques without explainable AI

S. M. Alzahrani [21] proposed a transformer model combining global and local levels of attention mechanisms for classifying brain tumors. Their model got entire dataset’s global discriminative features and correlations using external attention. Then they achieved image patche’s local discriminative features and correlations through the self-attention mechanism. Additionally, the mixers of depth and point-wise convolution highlighted key areas of spatial and channel-wise features of Images. Lastly, final output feature maps were achieved by the use of a squeeze-and-excitation mechanism. Furthermore, they used data-augmentation techniques with ConvAttenMixer by using rescale and data augmentation layers. Data augmentation and the simple classification head enhanced the model’s performance generally. For model evaluation, the dataset containing 7022 MRIs of the brain with 4 classes was used. Their model demonstrated an accuracy of approximately 97.94%.

In another study I. Gupta et al. [22], introduced an approach of 7-layered CNN to perform brain MRI classification in two classes as benign or malignant. They performed their investigation on a dataset containing 253 MRIs of the brain. For image preprocessing, the images were cropped to get the brain section only with the use of Edge Detection with Computer Vision (CV). The technique of data augmentation was used on the training dataset, using flipping, rotation, brightness, shear, and shift techniques to augment the amount of train data. They compared the efficiency of their proposed CNN model with some pre-trained models including ResNet-50, Inceptionv3, VGG-16 and some previous state-of-the-art models. Among these architectures, their proposed CNN model outperformed other architectures with 94% accuracy.

F. E. AlTahhan et al. [23] initially conducted classification using GoogleNet and AlexNet, two convolutional neural networks that were pre-trained and fine-tuned. Among these, AlexNet demonstrated superior performance. To further enhance the performance of fine-tuned AlexNet, they explored two hybrid approaches: one combining AlexNet with SVM and the other combining AlexNet with KNN. They worked with a limited dataset comprising 2880 brain MRIs (T1-weighted contrast-enhanced) and utilized K-Fold cross-validation to partition the data. The models, which included fine-tuned GoogleNet, fine-tuned AlexNet, AlexNet-SVM, and AlexNet-KNN, achieved accuracies of 88%, 85%, 95%, and 97%, respectively.

Z. Rasheed et al. [24] introduced a methodology starting with image enhancement techniques, including sharpening with Gaussian blur and applying Adaptive Histogram Equalization using CLAHE. They subsequently employed a modified CNN model for classification. Their study utilized an MRI dataset consisting of 7023 images for model training. Their approach was evaluated by comparing its effectiveness against pre-trained models including VGG19, InceptionV3, VGG16, ResNet50, and MobileNetV2. During experimentation, their method achieved 97.84% accuracy.

O. Özkaraca et al. [25] Firstly, basic CNN architecture, VGG16Net and DenseNet architectural structures were investigated to know the effect of transfer learning approaches on the success rates of their classification problem. Their findings showed that the transfer learning approaches that they used did not provide the results as expected. So, they proposed a modified CNN architecture and used 10-fold cross-validation by which they achieved 96% accuracy. They used 7021 brain MRIs for model training.

M. A. Gómez-Guzmán et al. [26] proposed a generic convolutional neural network (CNN) model. They also performed a study on six pre-trained CNN models. They evaluated their model with the dataset containing 7023 MRIs. The dataset was preprocessed by resizing the images and adding labels, also augmentation on the dataset was applied by Zooming or Scaling, Rotating, and adding Brightness to MRIs. They evaluated their proposed CNN, Xception, MobileNetV2, ResNet50, InceptionV3, Inception, ResNetV2, and EfficientNetB0. Among all the listed models the best performance was of InceptionV3, as it achieved 97.12% average accuracy.

C.C. Peng and B.H. Liao [27] introduced a convolutional neural network (CNN) approach for the classification of brain tumors from MRI images. Their dataset, sourced from Kaggle, consisted of 3264 MRIs categorized into four classes. Prior to input into the CNN architecture, the images were resized to 224 $\times$ 224 pixels. The CNN architecture comprised 24 layers designed for feature extraction. They reported an average classification accuracy of 94.4% on the testing set.

R. Imam and M. T. Alam [28] investigated the influence of some loss functions, such as focal loss, and also the methods for data oversampling, including ADASYN and SMOTE. Additionally, they also incorporated data augmentation in addressing the issue of data imbalance. The dataset that they used contained 4200 brain MRIs. They augmented samples of minority class from the dataset with contrast, brightness, and sharpness alteration each with 80% to 120% random intensity. They evaluated DenseNet201-CNN, EfficientNetB0-CNN, EfficientNetB3-CNN, GoogleNet-CNN, MobileNet-CNN, ResNet50-CNN, VGG16-CNN, and XceptionNet-CNN. With augmentation, their proposed approach, which integrates VGG-16 with CNN, reached 96% accuracy which was the highest among loss functions and oversampling methods.

M. M. Islam et al. [29] employed transfer learning models such as DenseNet121, InceptionV3, MobileNet, and VGG19. They utilized a Kaggle dataset consisting of 7023 brain MRIs categorized into 4 classes. Image augmentation for class balance was conducted using Keras’ ImageDataGenerator class. The study findings indicated that MobileNet achieved the highest accuracy of 99.60%.

S. Shanjida et al. [30] developed a novel CNN-KNN architecture for the detection and classification of various types of tumors. They utilized a dataset of 2879 MRIs from Kaggle, is categorized into four classes. As a preprocessing step, the images were resized and transformed into grayscale. The CNN was employed to extract different intensity-based features during the feature extraction phase. Subsequently, two machine learning classifiers, Softmax and KNN, were utilized in the classification step. KNN demonstrated superior accuracy compared to Softmax. Overall, their method achieved an accuracy of 95.7%.

Y. Arora et al. [31] proposed a multiclass brain tumor classification model using a weighted least squares twin support vector machine with fuzzy concepts. It improves traditional models by efficiently distinguishing support vectors from noise using membership and non-membership weights, local neighborhood information, and a fuzzy hyperplane. The model solves linear equations for computational efficiency. It achieves a precision of 93. 45% in the classification of four classes of brain tumors, demonstrating robustness and generalization. However, its reliance on fuzzy hyperplanes and parameter tuning may impact performance and interpretability, posing challenges in medical applications that require explanation.

W. Ayadi et al. [32] proposed a deep CNN model for brain tumor classification, addressing the challenges of manual MRI analysis, which can be time-consuming and error-prone. Accurate classification is crucial due to the varying location, texture, and shape of brain tumors. The model uses multiple layers in the CNN architecture and demonstrates superior performance on three datasets compared to existing methods. However, its effectiveness is based on large labeled datasets, which are difficult to obtain. Furthermore, the complexity of the model can lead to overfitting with limited data, and its lack of interpretability poses challenges to trust in medical applications.

S. Tummala et al. [33] examined an ensemble approach with multiple Vision Transformer (ViT) models to classify brain tumors based on T1-weighted contrast-enhanced MRI scans. The authors customized several pre-trained models ViT B/16, B/32, L/16, and L/32 from ImageNet. They tested their results on a Figshare dataset with 3,064 labeled MRI slices containing meningioma, glioma, and pituitary tumor. ViT L/32 model achieved the best test accuracy among single models with 98.2%, while the ensemble model of all four provided the best accuracy score of 98.7% at the end test. The accuracy achieved confirmed the utility of ViT models in the analysis of medical images for the classification of brain tumors; nonetheless, the deployment of these models in real-life clinical settings is challenged due to the enormous amounts of data and computational resources needed for training and inference, despite the precision they offer.

N. Aziz et al. [34] examined the application of deep learning models in automating brain tumor classification with the DenseNet architecture. The authors used the same Figshare dataset and performed transfer learning with four pre-trained models: DenseNet, ResNet, EfficientNet, and MobileNet. DenseNet achieved the highest test accuracy out of the other models at 96%, surpassing ResNet’s (91%), EfficientNet’s (91%), and MobileNet’s (93%) accuracy. To improve results, the authors applied regularization techniques such as data augmentation, dropout, batch normalization, and hyperparameter tuning to the fine-tuned DenseNet. With these changes, the model’s accuracy increased to 97.1%, indicating its capability for reliable diagnostic use. Nonetheless, despite these impressive results, the high dependence on pre-trained models and fine-tuning poses problems for collection generalization to other datasets.

### Deep learning techniques with explainable AI

K. V. Kumar et al. [35] proposed a framework for brain tumor classification using VGG19 and InceptionV3 CNNs, incorporating data augmentation techniques. They utilized a Kaggle dataset consisting of 253 MRI scans is categorized into two classes. The MRI scans underwent preprocessing steps including normalization, thresholding, cropping, and resizing to 229 $\times$ 229 pixels. For augmentation, they employed Keras’ ImageDataGenerator class to apply rescaling, horizontal and vertical shifting, shear transformation, zooming, rotation, flipping, and brightness adjustments to the images. The weights of the pre-trained VGG19 and InceptionV3 models were frozen, and the model architecture was extended by adding a flatten layer, a dense layer, and a sigmoid activation function to the sequential model. VizGradCAM was used for visualizing and interpreting the model’s predictions. Their experiments demonstrated high accuracy rates of 98% for VGG19 and 96% for InceptionV3 CNNs.

H. Benyamina [36] performed binary classification of brain tumors using a pre-trained VGG16 model. MRI dataset from Kaggel containing 3000 MRI images of two classes was used for brain tumor classification. XAI method SHAP was used for model explainability.

F. Ahmed [37] conducted a study using the VGG16 deep learning model to classify brain MRI images from a Kaggle dataset consisting of two classes: normal and tumor. The VGG16 model was trained and achieved a testing accuracy of 97.33%. To address the interpretability of deep learning models, Layer-wise Relevance Propagation (LRP) was applied to the VGG16. After training and making predictions with VGG16, LRP, an explainable artificial intelligence (XAI) method was employed to provide explanations for the model’s predictions. If the explanations provided by LRP were considered satisfactory, the trained model was deployed in the cloud. However, if the explanations were found inadequate, the model underwent retraining to enhance its performance.

L. Gaur et al. [38] introduced an explanation-driven deep learning model employing a convolutional neural network (CNN) with a dual-input strategy. They utilized a dataset comprising 4 categories totaling 2,870 images. Initially, all 512 $\times$ 512 $\times$ 3 images were resized to 150 $\times$ 150 $\times$ 3 pixels. Gaussian noise with a 0 mean and 100.5 standard deviation was applied for data augmentation to enhance accuracy. Their CNN model consisted of six hidden layers with an output layer size of 1 $\times$ 4. To enhance accuracy, the researchers utilized two copies of the dataset in the CNN, processing one through the convolution layer and the other through the fully connected layer. They performed K-fold cross-validation with K=10 non-overlapping folds across 20 epochs, 128 as batch size. For model interpretation, they used LIME and SHAP to interpret their predictions related to brain tumors. The trained CNN achieved 94.64% training accuracy and 85.37% test accuracy.

F. Mercaldo [39] introduced convolutional neural networks along with class activation mapping for explainability. They experimented with four distinct models: VGG16, ResNet50, Alex_Net, and MobileNet. They evaluated their approach on a dataset consisting of 3,000 images categorized into 2 classes, achieving an accuracy of 99% with the ResNet50 model. Prediction explanations were provided using Grad-CAM. To summarize the literature on existing techniques based on Deep Learning with and without XAI, Table 2 presents the numerous investigations that have been conducted on brain tumors in recent years.

**Fig. 1:**
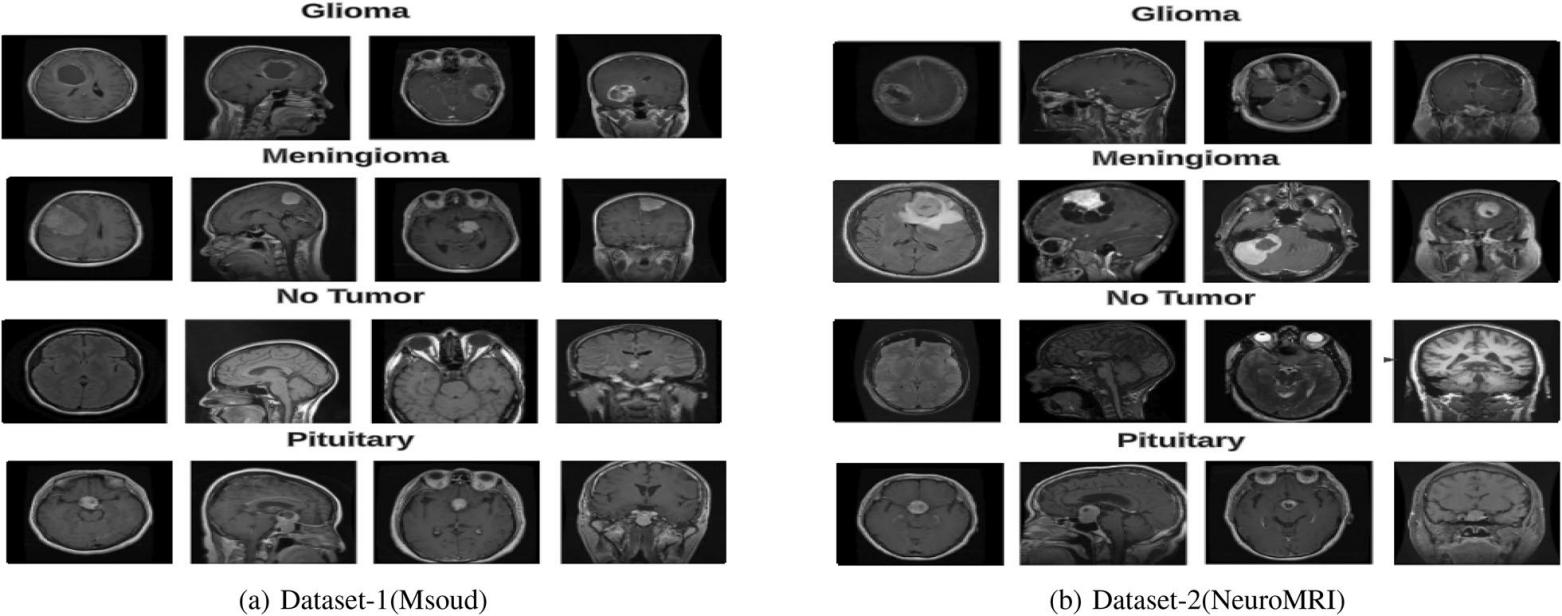
Sample MRIs from Msoud and NeuroMRI Datasets

## Material and methods

### Dataset description

During this process, two freely accessible brain MRI datasets were obtained. The first brain MRI dataset is the Msoud dataset of tumors that was constructed by Nickparvar (2021). This dataset includes 7023 brain MRIs, available in grayscale with JPG formats. The second dataset is NeuroMRI which is an open repository for detection and classification tasks. This dataset includes 3264 brain MRIs, available in grayscale with JPG formats. Table 3 displays the class distribution of both datasets, Both datasets consist of four distinct classes that are Meningioma, No-tumor, Glioma, and Pituitary displayed in Fig. 1.

**Table 3:** Description of Brain Tumor Datasets

Tumor type	No of MRIs (Msoud Dataset)	No of MRIs (NeuroMRI Dataset)
Meningioma	1645	926
No Tumor	2000	500
Glioma	1621	937
Pituitary	1757	901

### Dataset pre-processing

Data preprocessing is an important process of preparing the raw data for analysis or for training the model [40]. Before data preprocessing, the images in both datasets varied in size and were not consistently focused on the brain section, presenting challenges for effective analysis. Thus, differences in MRI acquisition settings, resolutions, and areas of focus in both datasets lead to domain shift, which affects generalization. To address these issues, both datasets underwent a thorough preprocessing procedure explained below:

1. Cropping: Images were first converted to grayscale and thresholded to separate the object from the background. Morphological operations (erosion and dilation) were applied to remove noise. The largest contour was found, and the extreme points (bottom-left, bottom-right, top-left, top-right) were used for cropping the original image and a few extra pixels were added to the cropped region.
2. Normalizing: The cropped images were normalized to ensure pixel values were within the range [0, 255], suitable for display and further processing.
3. Resizing: To reduce the computational cost, images were resized from 512 $\times$ 512 $\times$ 3 to 224 $\times$ 224 $\times$ 3, improving processing speed and reducing the load on deep learning models.

These preprocessing steps help reduce dataset biases by standardizing image quality, ensuring uniformity in brain region focus, and mitigating variations in image acquisition.

### Proposed methodology

**Fig. 2:**
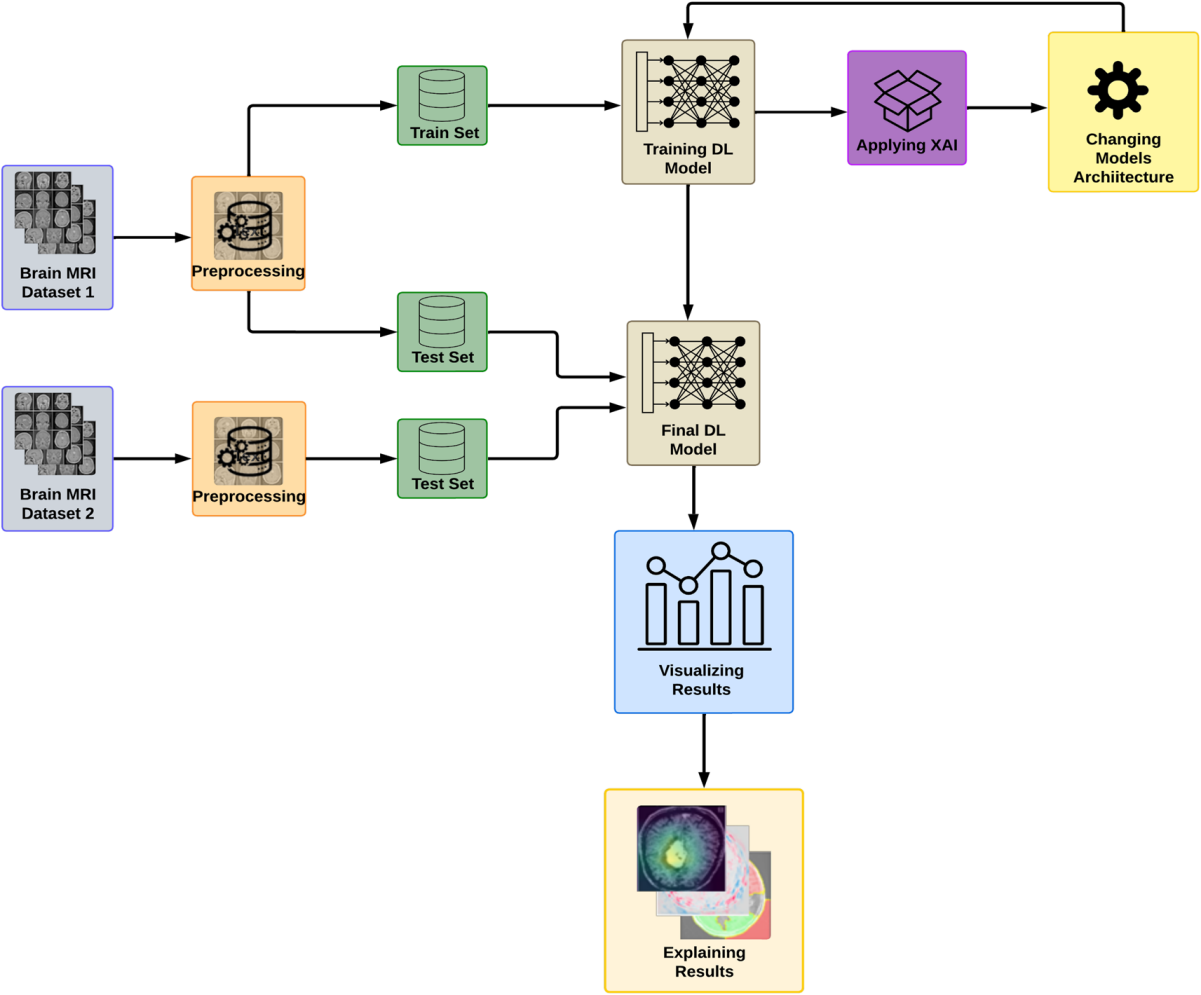
XAI Based Approach for Brain Tumor Classification

This research study presents a novel XAI-based CNN model, which is specifically designed for the task of Multi-Class Image Classification focusing on important features. By integrating XAI with CNN, our approach focuses on contributing abnormal soft tissues while also enabling the model to have a reduced number of layers, making it a valuable tool for various classification tasks of images. Figure 2 illustrates the proposed methodology.

#### CNN

The architecture of CNN is designed to emulate the visual processing of the human brain, consisting of key components such as convolution layers, activation functions, pooling layers, and fully connected layers. Convolution layers apply filters to input images to generate feature maps that highlight edges, textures, and patterns. ReLU and other activation functions presents non-linearity, enabling models to capture complex patterns. Pooling layers help decrease the spatial dimensions of feature maps, which in turn reduces computational demands and reducing over-fitting by retaining significant features. After several convolution and pooling stages, the abstract features are passed to fully connected layers, which combine them to make final predictions. In classification of multiple classes, The last layer employs a softmax activation function to generate a probability distribution that indicates the likelihood of each class.

**Fig. 3:**
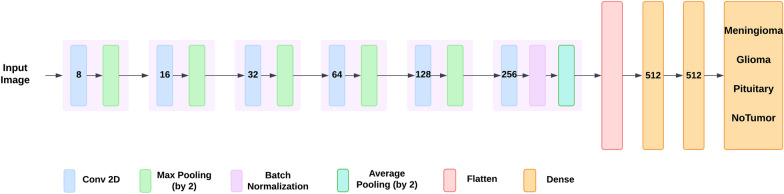
CNN Architecture

Our CNN model, designed for brain tumor classification, comprises multiple layers that process the input data in sequence. The model begins with a convolution layer that takes in 224x224x3 RGB images as input, applies 8 filters with a kernel size of 3x3, and then passes through a ReLU activation function. Next, a max pooling layer follows this layer that performs the down-sampling of the feature maps by a factor of 2 in both width and height. This sequence of convolution and max pooling layers is iterated several times, with increasing number of filters as 16, 32, 64, 128, and 256, and the kernel size remaining constant at 3x3. The padding is set to ’same’ to maintain the feature maps’ spatial dimensions. After the convolution and pooling layers, the model adds a batch normalization layer to normalize the activations, followed by an average pooling layer that down-samples the feature maps again. The output of the convolution and pooling layers is passed onto flatten layer which flattened it into a 1D feature vector. This vector is then directed into two dense layers with 512 neurons each and ReLU activation. In the final stage, the model includes a dense layer, which uses the softmax activation function to output a probability distribution over four classes. The CNN model can viewed in Fig. 3.

As brain tumor classification models complex structure often makes them difficult to interpret. This leads to challenges in understanding decision-making processes o classification which can make models rely on unnecessary features or normal soft tissues. Reliance on irrelevant features or normal soft tissues can negatively impact the performance of brain tumor classification models by reducing accuracy and increasing the likelihood of false positives or false negatives. When a model focuses on non-tumor regions, it may misclassify healthy tissue as a tumor or fail to detect actual tumor regions, leading to unreliable predictions. This misinterpretation can affect clinical decision-making, delaying proper diagnosis and treatment. This targeted approach directly addresses the limitations of current models that do not base their classifications on the actual tumor regions, leading to a significant improvement in diagnostic accuracy and reliability.

**Fig. 4:**
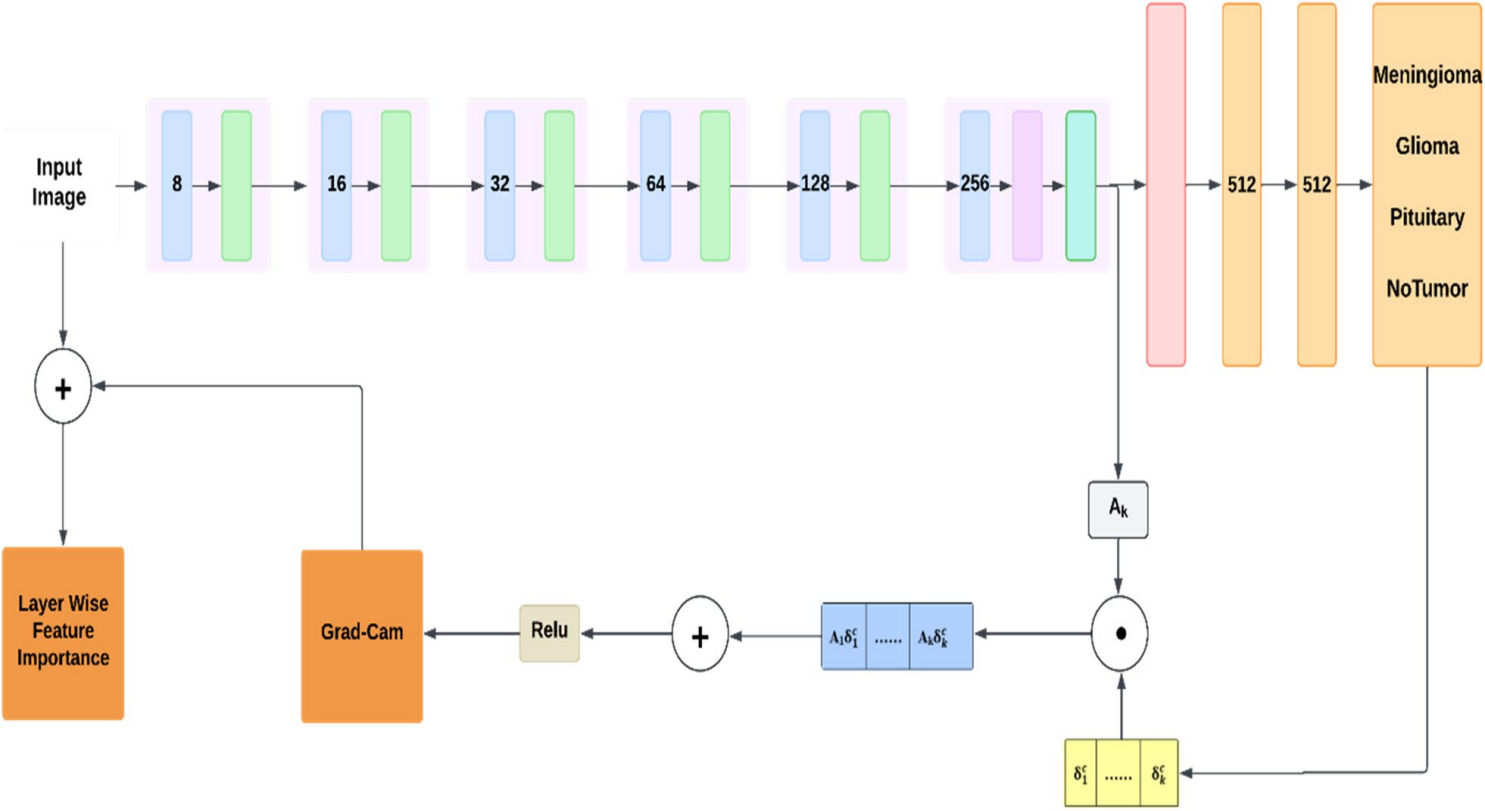
Proposed XAI Based CNN Architecture

Additionally, brain tumor classification models can have additional layers and parameters that make no difference in output but make it complex, and may produce imprecise results. To overcome these limitations, we used the XAI method Grad-Cam with CNN which made the model focus on important or tumorous features with a reduced number of layers as illustrated in Fig. 4. Simplifying a model without sacrificing accuracy of the model reduces complexity and time taken to train, making it more efficient and deployable in a resource-limited clinical setting.

#### Grad cam

Grad-CAM (Gradient-Weighted Class Activation Mapping) is an XAI method used to understand the importance of different layers and features within a CNN. Grad-CAM helps to locate the regions of an image having the greatest impact on the predictions. By examining different layers of the network, you can gain insights into the hierarchical importance of features at various model depths.

Grad-CAM [41] represents a progression from conventional CAM methods [42]. Unlike CAM, which relies on a CNN structured around global average pooling (GAP) [43], Grad-CAM is adaptable across various CNN architectures. In contrast to typical pooling methods, GAP compresses each 2D feature map into a 1D vector by averaging, which is then used for classification through the softmax function. The GAP operation is mathematically defined as shown in Equation (1).

1 $$\begin{aligned} g_p = \frac{1}{H \times W} \sum _{i=1}^{H} \sum _{j=1}^{W} u_{p(ij)} \end{aligned}$$

where $g_p$ represents the $p^{th}$ one-dimensional feature obtained after the GAP operation, with H and W denoting the height and width of the two-dimensional feature map, respectively. Additionally, $g_p$ corresponds to the $p^{th}$ convolved feature map located at position (i,j). The model’s attention map is created by integrating the convolved feature maps with the weights linking the GAP layer to the output, as detailed in Equation (2).

2 $$\begin{aligned} M_{(x,y)}^p = \sum _{j} w_j^p A_{(x,y)}^j \end{aligned}$$

where $M_{(x,y)}^p$ represents the class activation map for category p, $w_j^p$ denotes the weight of the $j^{th}$ feature map and $A_{(x,y)}^j$ is the $j^{th}$ convolved feature map located at position (x,y). To overcome CAM’s limitations, Grad-CAM adapts the approach to be applicable across different CNN architectures. Initially, it computes the output score for each category as follows:

3 $$\begin{aligned} S_p = \sum _{i=1}^{H} \sum _{j=1}^{W} w_j^p A_{(i,j)}^j \end{aligned}$$

where $S_p$ is the score for category p, H and W represent the height and width of the feature map, $w_j^p$ is the weight associated with the $j^{th}$ feature map for category p, and$A_j$ refers to the $j^{th}$ feature map. To obtain the category-specific positioning map, the gradient between the output score $S_p$ and the feature map $A_j$ is computed to acquire the category-specific positioning map:

4 $$\begin{aligned} \delta _j^p= & \frac{\partial S_p}{\partial A_j} \end{aligned}$$

5 $$\begin{aligned} G^{p}= & ReLU\left( \sum _{j} \delta _{j}^{p} A_{j}\right) \end{aligned}$$

where $\delta _p^j$ represents the weight of $j^{th}$ feature map, while $G_p$ represents the normalized heat map for category p.

In Proposed XAI-based CNN architecture during the forward pass, the images were passed through the CNN architecture to extract various features. After the forward pass, the network provided an output, which is the prediction of the tumor type. Once the prediction is made, a backward pass is performed to calculate the gradients of the output (specifically, the score for the predicted class) with respect to the feature maps of a chosen convolutional layer. The gradients were averaged over the width and height dimensions of the feature maps. The feature maps were then multiplied by their corresponding importance weights. The weighted feature maps were summed to produce the Gradient activation mapping, highlighting the regions of the image that were most important for the classification. The ReLU activation function was applied to this output to retain the features that positively influence the model’s decision. This output was then combined with the input image to get the layer-wise feature importance. These steps were applied across all layers to assess the feature importance at each layer. Through this process, it is determined which layers contribute most significantly to the model’s decision.

#### Modifying models architecture

Based on the analysis achieved from Grad-Cam the CNN models’ architecture was modified. The mean of all the layer’s importance was calculated and saved. To remove the layers that are not contributing much to the model’s performance, a threshold was set and layers below that threshold were removed from the model. This threshold was the least mean value among all the layer’s mean values. After removing those layers the model underwent the training phase again.

### Model explainability

We employed the Explainable Artificial Intelligence (XAI) methods to interpret the model’s decision-making processes and make classification process understandable and transparent. Explainable AI (XAI) incorporates mechanisms to make the AI model’s decisions are transparent, allowing users to understand how and why decisions are made. Transparent models ensure that decisions are based on clinically relevant features, minimizing the risk of errors from relying on irrelevant patterns. By making AI predictions easier to interpret, clinicians can validate and enhance their assessments with insights from AI, resulting in better diagnostic accuracy. Transparent decision-making aligns with healthcare regulatory requirements, ensuring that AI models adhere to ethical and legal standards for medical use. XAI encompasses various techniques and approaches that can be generally categorized into several types, depending on their methodology and application:

1. Post-Hoc Explainability techniques analyze models after training. Feature importance methods like permutation importance and gradient-based importance identify influential features. Saliency maps and heatmaps highlight important image areas. LIME explains individual predictions by perturbing inputs, while SHAP uses game theory concepts to measure feature importance.
2. Intrinsic Explainability involves inherently interpretable models. Decision trees use simple feature splits, linear and logistic regression models provide feature coefficients, and rule-based models use if-then rules for decision-making.
3. Model-Specific Explainability techniques are tailored to specific models. Attention mechanisms in transformers show which input data the model emphasizes. Feature visualization, like activation maximization, displays input patterns that activate specific neurons or layers.
4. Model-Agnostic Explainability methods apply to any AI model. Partial dependence plots (PDPs) show the impact of features on predictions, and surrogate models use simple models to approximate complex model behavior.
5. Global Explainability techniques provide an overall model understanding. Feature importance ranking identifies impactful features, while global surrogate models approximate the entire model’s behavior.
6. Visualization Tools use visual aids to illustrate the model’s decisions. Heatmaps and saliency maps highlight important data regions, and decision plots and graphs illustrate feature impact on predictions.

In our research study specifically, we used Grad-CAM, SHAP, and LIME to enhance the interpretability of our CNN-based brain tumor classification model, with Grad-CAM selected as the primary XAI method due to its superior spatial localization capabilities. Grad-CAM effectively highlights abnormal tumor regions in MRI scans, ensuring clinically relevant interpretations, whereas SHAP provides feature importance analysis without precise spatial localization, and LIME offers instance-based explanations that may vary across predictions. Given the need for robust, spatially precise tumor identification in medical imaging, Grad-CAM was chosen as the most suitable method, while SHAP and LIME were used for additional validation.

#### SHAP

SHAP (Shapley Additive explanations) is a model-agnostic XAI technique. It allocates a value to each feature of an input instance for a given prediction, indicating its individual contribution to the output. This approach is grounded in Shapley values originated from game theory, which are employed to equitably share total gains among participants in cooperative games [44].

SHAP estimates the contribution of every feature to the forecasted result by simulating the absence of that feature. This is done by creating multiple versions of the input instance, each with a different feature missing or replaced with a reference value. The model is then evaluated on each of these modified instances, and the variation in the predicted result is used to estimate the contribution of the missing feature as described in the below algorithm.

**Algorithm 1:**
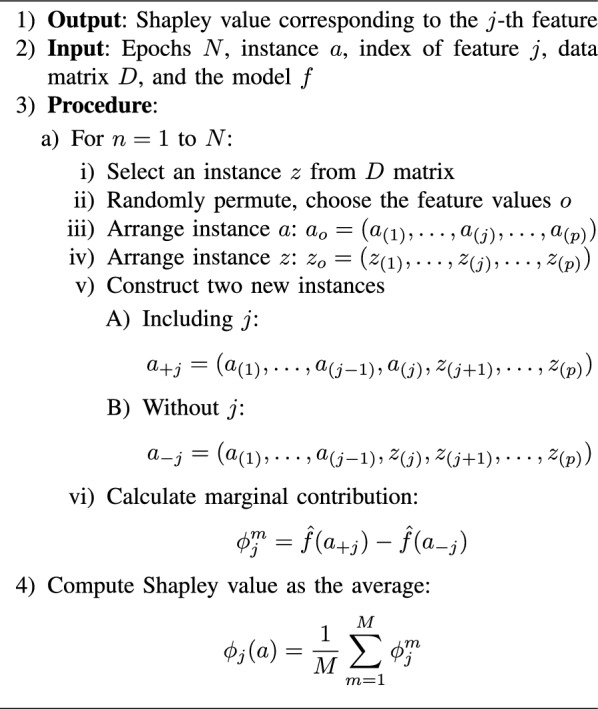
Algorithm for Shapley Value

**Table 4:** Key Hyperparameters for Experimental Setup

Parameter	Value
Optimizer	Adam
Learning rate	0.001
Loss function	Sparse categorical cross entropy
Batch size	40
Epochs	40

SHAP is used for both tabular and image data. Explaining images with SHAP involves applying the same principles used in tabular data but adapted to the peculiarities of the image data. The core idea is still based on Shapley values, which allocate the contribution of each pixel (or group of pixels) to the final prediction made by an image classification model.

#### LIME

By approximating, LIME (Local Interpretable Model-agnostic Explanations) explains the outcomes of complex models locally around a specific instance with an interpretable model. For images, LIME generates explanations by perturbing the image, analyzing the resulting changes in the model’s predictions, and fitting a simple model to these observations [45].

LIME works by first segmenting the images into superpixels, which are contiguous regions of pixels that share similar characteristics. This reduces the complexity of the image and allows LIME to handle a smaller number of features (superpixels) instead of individual pixels. After that LIME generates several perturbed versions of the original image. This is done by randomly turning superpixels on and off. Turning a superpixel off typically means replacing it with a baseline value, such as the average color or black pixels. Each perturbed image is thus a simplified version of the original image with certain superpixels removed or modified.

Class probabilities for each perturbed image are then predicted using the original model. This generates a set of prediction scores corresponding to the perturbed images. For each perturbed image, a binary vector is created indicating which superpixels are present (turned on) or absent (turned off). This matrix, along with the corresponding predictions, serves as input for the local surrogate model. LIME weights perturbed images based on their similarity to the original image. A common approach is to use a kernel function to compute these weights, giving higher weights to perturbed images that closely resemble the original image. The proximity measure is represented as:

6 $$\begin{aligned} w_i = \exp \left( -\frac{D(x, z_i)^2}{\sigma ^2}\right) \end{aligned}$$

where $D(x, z_i)$ is the distance between the original image x and the perturbed image $z_i$, and $\sigma$ is a scaling parameter.

The binary perturbation matrix and the weighted predictions are used to train the interpretable model. This model approximates the original complex model’s behavior in the vicinity of the original image. The coefficients of the surrogate model explain. For the linear model, these coefficients indicate the significance of each superpixel in the model’s prediction for the original image. Positive coefficients indicate that the presence of a superpixel increases the likelihood of the predicted class, while negative coefficients indicate the opposite.

## Result analysis and discussion

### Experimental setup

The entire experiment was implemented in Python-3 using the TensorFlow framework on Kaggle Notebooks, which had specifications including 13GB of RAM and a 16GB P100 GPU. The dataset-1 training set, validation set, and testing set consist of 5618, 702, and 702 images with the ratio of 80%,10%, and 10%, and dataset-2 was only used to test the model. The other specifications are mentioned in Table 4. Hyperparameter selection was guided by empirical tuning and prior research. Learning rates of 0.01, 0.005, 0.001, and 0.0005 were tested, with 0.001 chosen for stable convergence. Batch sizes of 16, 32, 40, and 64 were evaluated, and 40 provided the best balance between stability and efficiency. The model was trained for 40 epochs, as further training yielded no significant improvement. The initial CNN model completed training in 86 s for 24 epochs, with an inference time of 10 ms per image, making it a fast and efficient baseline. However, after integrating XAI techniques, the model required 135 s for 40 epochs, with a slightly increased inference time of 11 ms per image. For model explainability Grad-Cam, Shap, and Lime are used. To assess our model’s classification performance, we utilized four important metrics: Accuracy, Precision, Recall, and F1 score. Following are their mathematical representations:

7 $$\begin{aligned} \text {Accuracy}= & \frac{TP + TN}{TP + TN + FP + FN} \end{aligned}$$

8 $$\begin{aligned} \text {Precision}= & \frac{TP}{TP + FP} \end{aligned}$$

9 $$\begin{aligned} \text {Recall}= & \frac{TP}{TP + FN} \end{aligned}$$

10 $$\begin{aligned} F_1= \ 2 \times \frac{\text {Precision} \times \text {Recall}}{\text {Precision} + \text {Recall}} \end{aligned}$$

where:

- *TP* = True Positives
- *TN* = True Negatives
- *FP* = False Positives
- *FN* = False Negatives

### Explainability results

The explainability evaluation begins by presenting the Grad-Cam results for both the initial and the proposed models. Following this, we illustrate the model’s explainability using the XAI methods SHAP and LIME. By utilizing this approach we addressed the shortcomings of earlier methods, which often lacked transparency and relied on unnecessary features for classification.

**Fig. 5:**
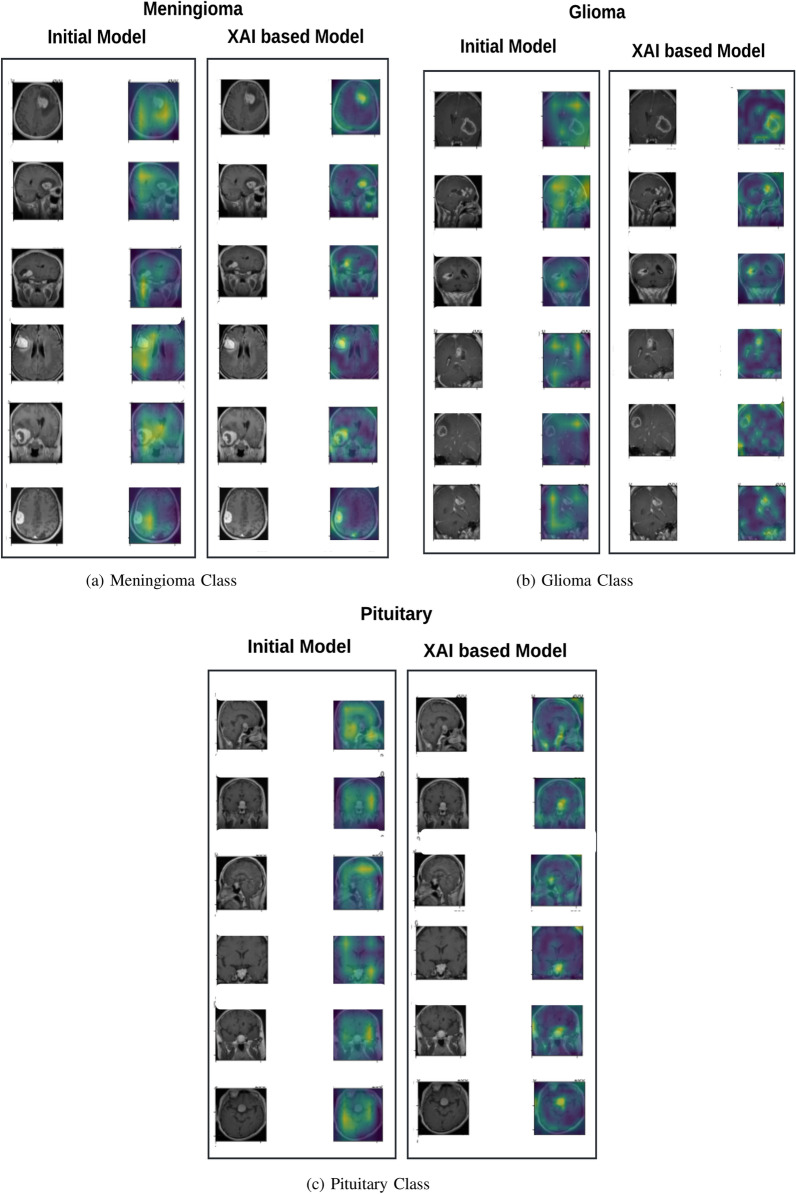
Grad-Cam Results

**Fig. 6:**
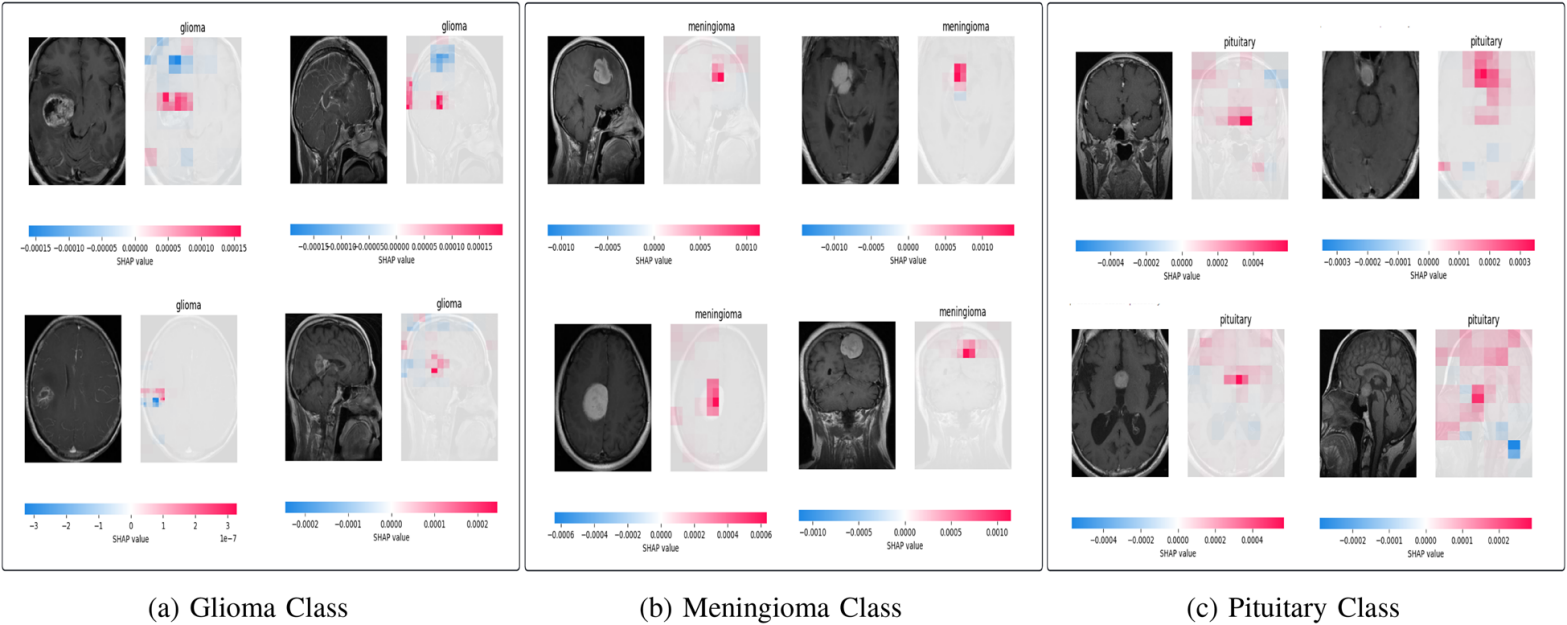
Shap Explanations

**Fig. 7:**
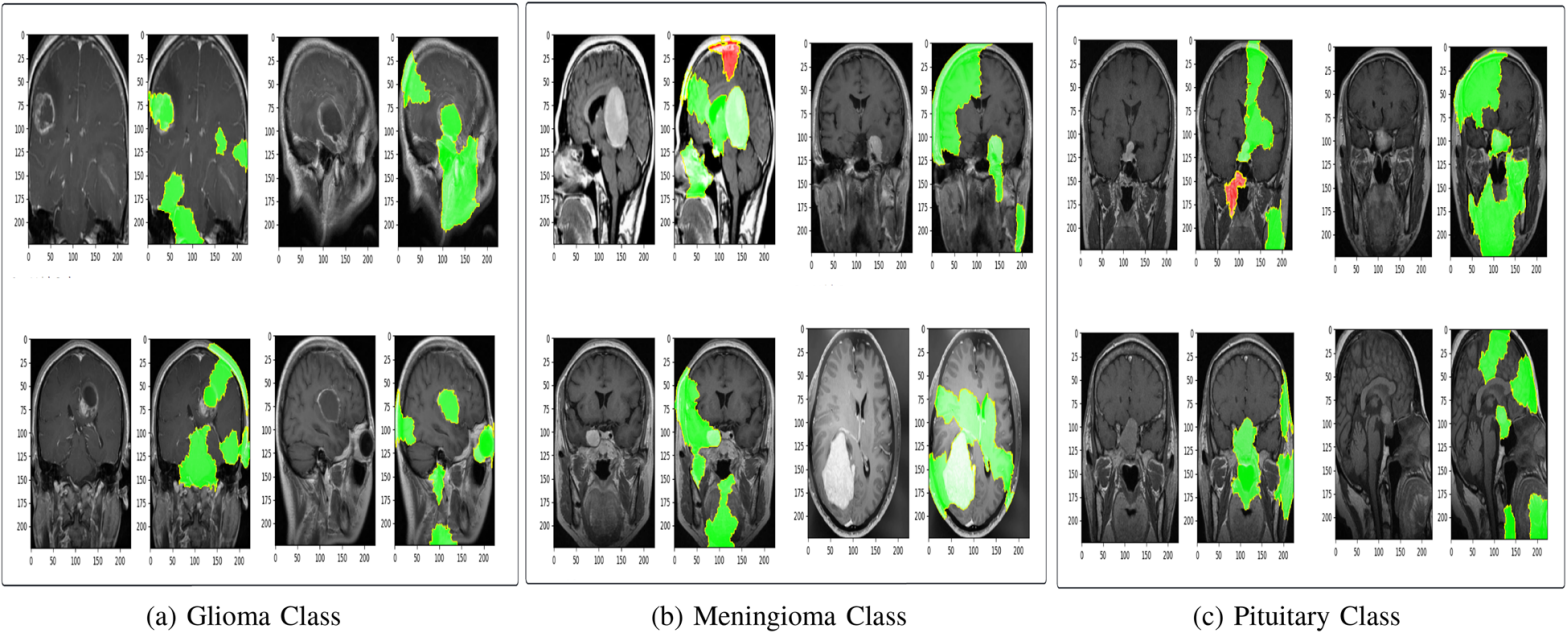
Lime Explanations

Grad-CAM enabled the visualization of brain regions of MRIs on which the model relied while making the predictions. The Grad-Cam results of the Meningioma, Glioma, and Pituitary classes are shown in Fig. 5. The attention of the initial model was dispersed in the case of meningiomas. However, the extra-axial regions lateral to the meninges where tumors develop were accurately captured by the XAI-based model. In gliomas, the initial model’s attention was at times directed towards the surrounding brain regions instead of the tumor. The XAI-based model correctly captured intra-axial regions with thick, irregular borders of the tumor and with central necrotic cores, which signify gliomas on MRI. For pituitary tumors, the initial model frequently and incorrectly assigned features of the adjacent structures. However, the XAI-based model accurately captured the abnormal growth of tissues in the sellar region, which is in keeping with the clinical presentation of pituitary adenomas.

The results obtained from SHAP and LIME show the specific features and brain regions that influenced the predictions of the model. In SHAP (Shapley Additive explanations), the visual representation uses colors to denote the level of features contribution to the model’s predictions as illustrated in Fig. 6. The features highlighted in red indicate significant positive contributions to the output prediction. However, the features highlighted in blue suggest minimal or negative contributions to the prediction. For gliomas, SHAP highlighted irregular tumor boundaries and necrotic core regions, which are known clinical indicators of high-grade gliomas. In meningiomas, SHAP-based heatmaps emphasized the extraaxial location along the meninges, aligning with their well-defined, homogeneous contrast-enhancing nature. In pituitary adenomas, SHAP correctly identified the sellar region and adjacent structures, crucial to distinguish pituitary tumors from other intracranial abnormalities.

In LIME (Local Interpretable Model-agnostic Explanations), the visual representation employs a color gradient to signify the level of importance of features for a specific prediction or instance as shown in Fig. 7. The features highlighted in green represent areas where the model’s prediction is positively influenced. In contrast, features highlighted in red indicate areas where the model’s prediction is negatively influenced or where the feature is not influential. For gliomas, LIME focused on regions infiltrated by tumors with heterogeneous intensity, strengthening its recognition of aggressive tumor characteristics. For meningiomas, the highlighted areas correspond to dural attachment and homogeneous contrast enhancement, aligning with their typical presentation on magnetic resonance imaging. In pituitary tumors, the model effectively identified the sellar region and its upward extension, which is well correlated with common clinical observations in macroadenomas.

**Table 5:** Comparison of Results with Initial and Proposed Model on Dataset-1 and Dataset-2

Dataset	Model	Accuracy	Precision	Recall	F1-Score
Dataset-1(Msoud)	Initial Model	98.95%	98.96%	98.90%	98.93%
	Proposed Model	99.21%	99.22%	99.18%	99.20%
Dataset-2(NeuroMRI)	Initial Model	93.33%	93.35%	93.83%	93.24%
	Proposed Model	94.72%	94.70%	95.10%	94.63%

**Fig. 8:**
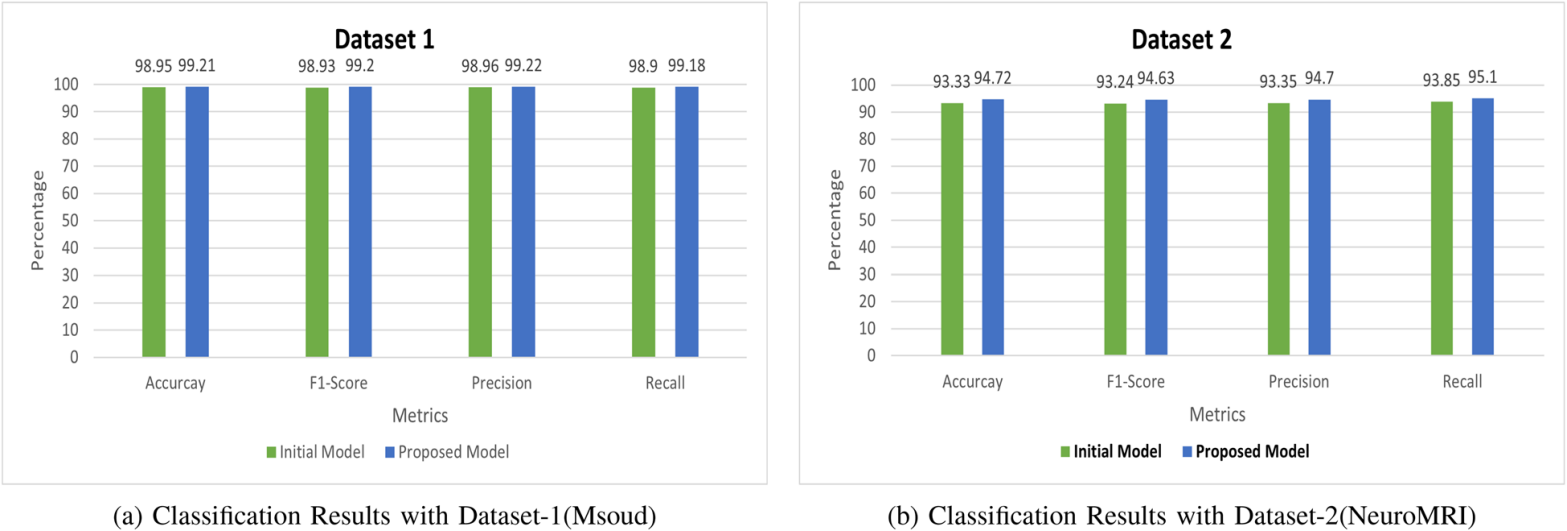
Bar Graphs Depicting Classification Results on Dataset-1 and Dataset-2

**Fig. 9:**
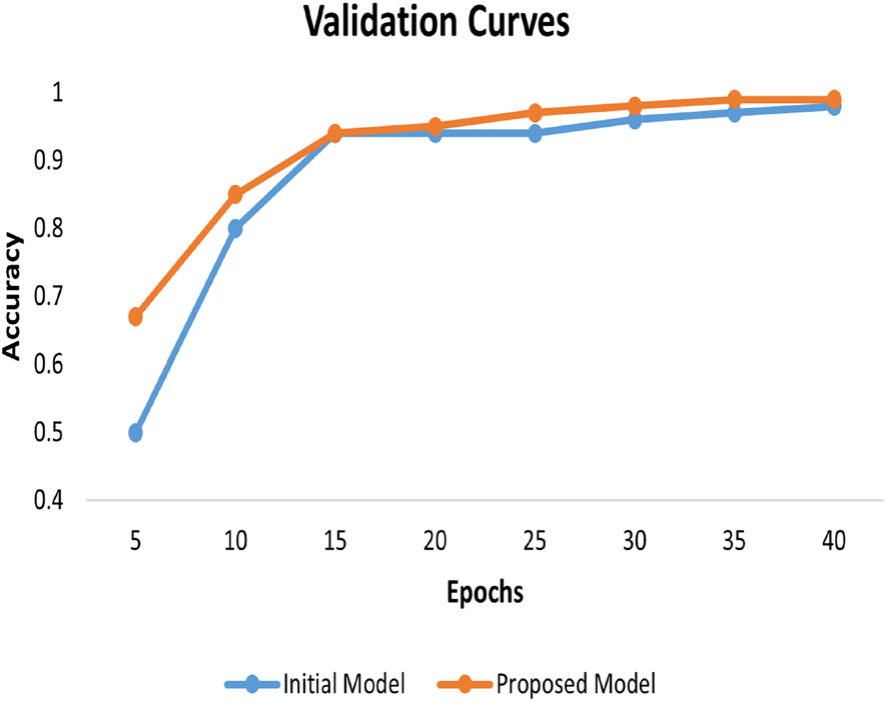
Learning Curves of Initial and Proposed Model

### Classification results

To evaluate the proposed model’s classification performance, we adopted four important metrics: Accuracy, Precision, Recall, and F1 score, along with their respective bar graphs. These metrics provide an extensive understanding of the model’s capability to differentiate among various classes. Finally, we display the accuracy curves for the proposed approach.

On Dataset-1, our model demonstrated outstanding performance, securing a notable accuracy of 99.21% as displayed in Table 5 and Fig. 8 (a). The model’s high performance level is emphasized by these results of precision and recall, which are critical for accurately classifying brain tumor subtypes. This implies the model is learning the distinguishing features of each subtype effectively, leading to enhanced performance in correctly identifying each category.

To test the model’s generalization ability, we evaluated its performance on an unseen dataset. Our proposed model achieved a notable accuracy of 94.72% on the new dataset as displayed in Table 5 and Fig. 8 (b). These results indicate that the model has efficiently learned the distinctive critical features for accurate classification of brain tumors. The model’s reliability is demonstrated by its efficiency in generalizing well to unseen data, which makes it valuable for practical medical applications where new and diverse data are frequently encountered.

**Table 6:** Comparison of Existing Techniques with Proposed Method

Source	Method	No of MRIs	Classification	XAI Method	Accuracy
[21]	Conv Atten Mixer	7023	Multiclass	–	97%
[23]	AlexNet-KNN	2880	Multiclass	–	97%
[24]	Image Enhancement + Modified CNN	7023	Multiclass	–	97%
[25]	Modified CNN	7023	Multiclass	–	96%
[26]	InceptionV3	7023	Multiclass	–	97%
[27]	CNN-24 Layers	3264	Multiclass	–	94%
[28]	VGG-CNN	4200	Multiclass	–	96%
[30]	CNN-KNN	2879	Multiclass	–	95%
[37]	VGG16	3000	Binary	LRP	97%
[38]	CNN with dual-input	2870	Multiclass	Lime and shap	85%
[39]	Resnet50	3000	Binary	Grad-cam	99%
Proposed	XAI based CNN	7023	Multiclass	Grad-Cam, Shap, and Lime	99%

To further evaluate the model’s performance we created learning curves for both the initial and proposed models shown in Fig. 9. These learning curves reveal how the models’ accuracy evolves over successive epochs. These curves indicate that the Proposed Model consistently outperforms the Initial Model, achieving better validation performance across all stages of training.

## Discussion and comparison

In this study, a novel XAI-based CNN method is presented for classifying brain tumors. The proposed CNN model processes 224x224x3 RGB images using a sequence of convolutional and max pooling layers with increasing filters (8 to 256) with ReLU activation, following batch normalization, average pooling, and dense layers, ultimately outputting a probability distribution across four classes using softmax. The initial model achieved a high classification performance. However, a significant challenge with the initial model was its lack of interpretability, making it difficult to understand which features should influence the model’s decisions making it rely on irrelevant features or normal tissues.

The proposed XAI-based model was designed to address these limitations by incorporating Grad-CAM. The importance of each layer was assessed by Grad-Cam, and layers less than a specific threshold were systematically removed, leading to a more efficient model without compromising performance. By systematically refining the model based on Grad-CAM analysis, we ensured that only the most important features were retained, leading to a more efficient and interpretable model. We compared the performance of an initial CNN model without XAI and proposed CNN model with XAI method Grad-CAM, to assess their performance.

The explainability of the proposed model was validated using Grad-Cam, SHAP, and LIME, which provided insights into the specific features that influenced the model’s predictions. Grad-CAM visually highlighted the brain regions, the model focused on during classification. By comparing the Grad-CAM visualizations of the initial and proposed models, we observed that our XAI-based model significantly improved its ability to accurately highlight and focus on abnormal brain tissues compared to the initial model. This improvement is achieved due to the XAI method guiding the model to prioritize the most relevant features. SHAP’s visual representations showed that features with significant contributions to the predictions of the model were primarily associated with abnormal brain tissues. LIME’s localized interpretations confirmed that the model’s decisions were influenced by important features, leading to accurate and explainable predictions.

In terms of classification performance, the proposed XAI-based model significantly outperformed the initial model, achieving an accuracy and F1-Score of 99.21% on dataset-1, highlighting its ability to accurately classify brain tumor subtypes. The generalization ability of the proposed model was also tested on an unseen dataset, where it maintained a strong performance with an accuracy and F1-Score of 94.72%. The improvement in classification performance and generalization ability of the XAI-based model over the initial model is due to its ability to learn and focus on the actual abnormal tissues responsible for differentiating tumor classifications.

The use of XAI techniques especially Grad-CAM, significantly improved the model’s ability to focus on and highlight abnormal brain tissues by guiding it to prioritize the most relevant features during training. This refinement was only due to the targeted approach provided by XAI, which helped the model better identify and emphasize critical regions in the input images. Although the model achieves 99% accuracy on seen data, a slight decrease in performance on new data is anticipated due to variability in the dataset. However, the minimal drop in accuracy indicates a strong generalization, making the model a reliable choice for real-world clinical applications.

Alongside Grad-CAM, the XAI methods SHAP and LIME were crucial in validating the model’s decisions and enhancing its interpretability. SHAP provided a global view of the feature importance, confirming that the model focused on relevant abnormal brain tissues, while LIME offered localized interpretations, ensuring consistency and explainability in the model’s predictions. This combination of global and local insights ensured the model was accurate, interpretable, and was not relying on irrelevant features or overfitting the data. This approach not only enhanced the model’s performance but also made it a valuable tool in the clinical diagnosis of brain tumors, where understanding the reasoning behind a prediction is as important as the prediction itself.

To further validate our results, we compared the performance of the proposed XAI-based CNN model with different related studies of brain tumor classification. This comparison is displayed in Table 6. The comparison of existing techniques with the proposed method reveals several key strengths of the proposed approach. Unlike many studies that focus on binary classification [37] and [39], the proposed method effectively handles multiclass classification. Furthermore, while many existing methods either lack XAI techniques [21, 23–28], and [30] or utilize only one or two XAI approaches [37, 39], the proposed method integrates multiple XAI techniques, including Grad-CAM, SHAP, and LIME. Additionally, the proposed method gained the highest accuracy of 99%, which surpasses the related studies including [21, 23, 24, 26, 37], and others. This highlights the success of the proposed approach in the domain of brain tumor classification.

We did not perform additional statistical tests because our focus is on ensuring that the model reliably captures tumorous regions for classification. Statistical tests primarily measure overall accuracy and do not reflect the deeper, clinically relevant differences in model behavior, where our XAI-based model clearly outperforms the initial model by prioritizing tumorous regions.

## Threats to validity

In this paper the model’s training and testing were conducted solely on magnetic resonance imaging (MRI) scans. Consequently, the findings may not be applicable to other imaging techniques, like CT scans, which could limit its effectiveness in practical applications. For enhancing the performance of the deep learning model, precisely the CNN model, the study utilized the explainable AI (XAI) method Grad-Cam. Additionally, methods like Shap and LIME were employed to test the model’s results. However, it is critical to note that not all possible XAI techniques were used in this study, and future research aims to incorporate more of these methods.

## Conclusion and future work

This paper presented an XAI-based approach for the classification of brain tumors which demonstrated significant results in accurately identifying and classifying different brain tumors subtypes by concentrating on critical features in the MRIs. The incorporation of XAI in the CNN architecture signifies an important advancement, enabling the model to make more informed decisions about feature emphasis. This enhancement improves classification accuracy and produces highly transparent and reliable models. Our dataset preprocessing strategies, Grad-CAM-based model, and use of XAI methods like SHAP and LIME for model interpretability collectively build a model that makes its classification decisions based on actual abnormal brain tissues.

Moreover, this study successfully identified the real contributing abnormal tissues from brain MRIs, significantly improving the overall performance of brain tumor classification. The model achieved interpretability, offering transparency into its decision-making process, which makes it superior to existing non-interpretable approaches. By providing insights into the model decision-making process, the study was able to reduce the number of layers in brain tumor classification models. The model demonstrated outstanding accuracy, precision, recall, and F1 score, achieving 99% on Dataset-1 and 95% on Dataset-2. These results suggest that the model is not only accurate but balanced in terms of precision and recall. Additionally, it outperformed several benchmark techniques by offering transparency and classifying MRIs based on crucial abnormal features with reduced layers.

In the future, there are several promising directions to explore. First, incorporating more diverse brain tumor MRI datasets could increase the efficiency of the model in generalizing to unseen data across different populations and tumor subtypes. Second, developing and testing real-time application capabilities for clinical settings will ensure that the model can provide instant, interpretable results. Third, exploring the integration of other imaging modalities, such as PET or CT, could create a more comprehensive diagnostic tool. And XAI techniques used, such as Grad-CAM, SHAP, and LIME with the proposed model, can be adapted for other medical imaging tasks, such as detecting lung diseases and identifying retinal abnormalities, as the proposed method ensures that the models focus on clinically relevant features. Additionally, this approach can be extended to classify and segment images in dermatology, radiology, and abdominal imaging, enhancing interpretability and reliability in clinical applications. Finally, investigating more advanced XAI techniques will further refine feature analysis and improve model interpretability. As future XAI techniques could track model decisions over time, enable interactive queries for customized explanations, and incorporate causal inference to reveal cause-and-effect relationships, enhancing diagnostic reliability. Additionally, advanced methods could measure prediction uncertainty, helping clinicians assess confidence levels and reduce overreliance on AI results.

## Data Availability

No datasets were generated or analysed during the current study
